# Biomedical applications of glycosylphosphatidylinositol-anchored proteins

**DOI:** 10.1194/jlr.R070201

**Published:** 2016-10

**Authors:** Susanne Heider, John A. Dangerfield, Christoph Metzner

**Affiliations:** Institute of Virology,* University of Veterinary Medicine, 1210 Vienna, Austria; Anovasia Pte. Ltd.,† Singapore 138623

**Keywords:** cancer, human immunodeficiency virus, immunology, lipid rafts, membranes, gene therapy, molecular painting, tumor therapy, viral envelope

## Abstract

Glycosylphosphatidylinositol (GPI)-anchored proteins (GPI-APs) use a unique posttranslational modification to link proteins to lipid bilayer membranes. The anchoring structure consists of both a lipid and carbohydrate portion and is highly conserved in eukaryotic organisms regarding its basic characteristics, yet highly variable in its molecular details. The strong membrane targeting property has made the anchors an interesting tool for biotechnological modification of lipid membrane-covered entities from cells through extracellular vesicles to enveloped virus particles. In this review, we will take a closer look at the mechanisms and fields of application for GPI-APs in lipid bilayer membrane engineering and discuss their advantages and disadvantages for biomedicine.

## GLYCOSYLPHOSPHATIDYLINOSITOL-ANCHORED PROTEINS IN BIOTECHNOLOGY: GENETIC VERSUS PROTEIN ENGINEERING

**Fig. 1.**
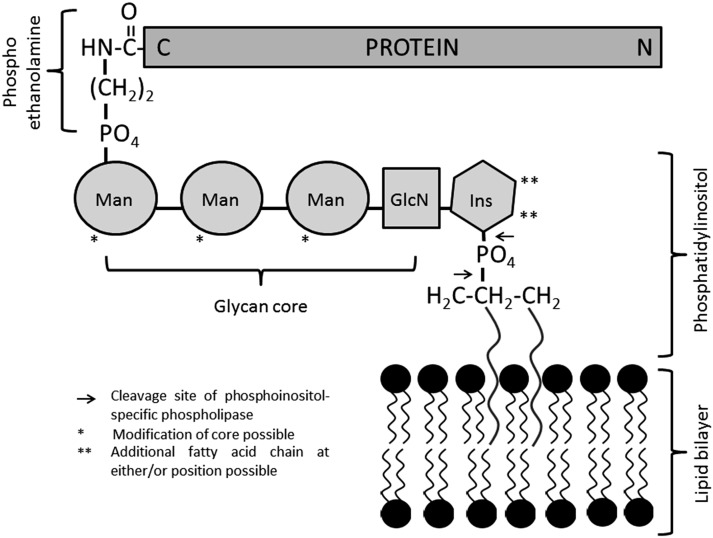
Structural features of GPI-APs. The C terminus of the protein is linked via phosphoethanolamine to the mannose (Man) core followed by glucosamine (GlcN) and the phospho inositol (Ins) carrying the lipophilic residues. Single asterisks indicate sites of additional side chains. Double asterisks indicate sites of a potential additional fatty acid moiety. Arrows indicate cleavage sites of phosphoinositol-specific phospholipases.

**Fig. 2.**
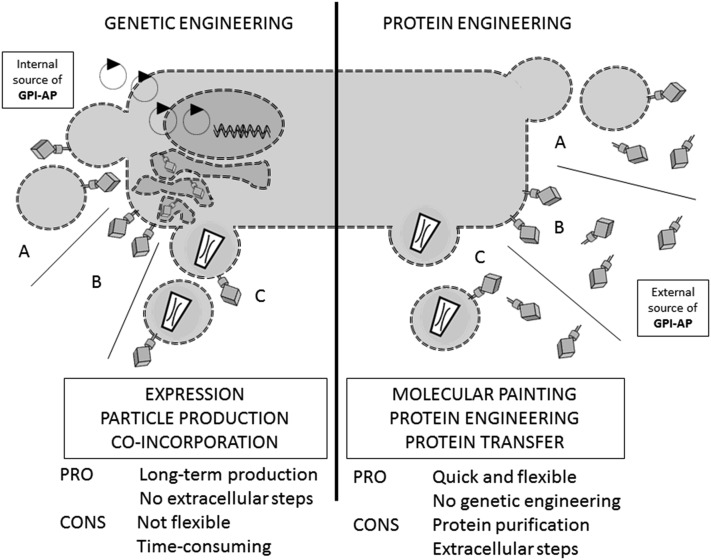
Overview of GPI-AP membrane engineering. Two different strategies are employed to modify lipid bilayer membranes with GPI-AP: GE (left) introduces recombinant DNA to express and display the proteins in cell membranes (B) and derived vesicles, such as virus particles (C) and exosomes (A). The vesicles receive GPI-APs as a result of co-incorporation during particle production. In PE (right) purified GPI-APs are inserted directly into the membranes of cells (B), virus envelopes (C), or membrane vesicles, e.g., exosomes (A), from an external source in a process termed, variably, PE, protein transfer, or MP. The advantages and disadvantages are briefly listed at the bottom of the figure. For more details see the Discussion, Summary, and Conclusions section.

**Fig. 3.**
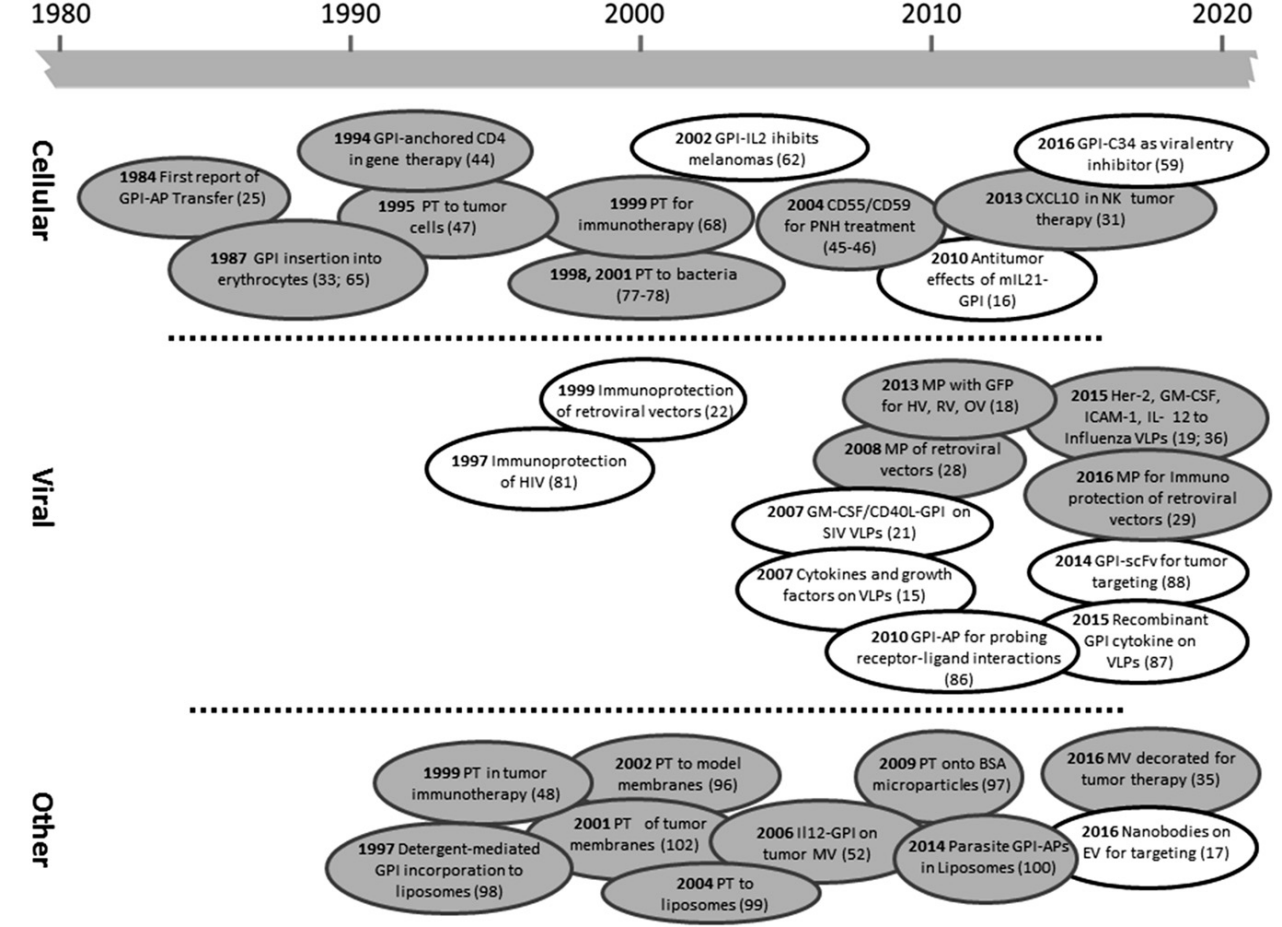
Landmarks in GPI-AP membrane engineering. The timeline depicts a selection of key developments in GPI-AP engineering of cellular (top), viral (middle), and other (bottom) lipid bilayer membranes facilitated by GE (clear bubbles) or PE (gray bubbles). EV, extracellular vesicles; HV, herpesviridae; MV, membrane vesicles; OV, orthomyxoviridae; PT, protein transfer; RV, retroviridae; scFv, single chain variable fragment. References to publications can be found in parentheses. For additional information on the proteins used, *see* Table 1.

Glycosylphosphatidylinositol (GPI)-anchored proteins (GPI-APs) are generated by posttranslational modification and can be found on approximately 0.5 percent of proteins in eukaryotes (1), while similar structures are also found in archaea (2). While the core structure of the GPI anchor is fairly conserved, i.e., a phosphoethanolamine linker located at the protein C terminus that is coupled to a glycan core mostly consisting of mannose residues, glucosamine, and inositol, which in turn comprise the head-group of the phospholipid (see **Fig. 1**), fatty acid residues can vary significantly as well as carbohydrate side chains. Proteins are singled out for GPI anchoring due to the presence of a GPI signaling sequence (GSS). The GSS contains the later site of GPI attachment (the amino acid in the ω position) and a series of hydrophobic amino acids, essentially forming a membrane-associating domain linking the pre-GPI protein to the luminal side of the endoplasmic reticulum. Biosynthesis of the anchor occurs separately and consists of a complex series of enzymatic reactions involving more than 11 enzymes (3). Synthesis starts at the cytosolic side of the endoplasmic reticulum with phosphoinositol, flips to the lumenal side, and sequentially adds the carbohydrate core elements. The transamidase enzyme complex replaces the GSS with the preformed GPI anchor by amide bond formation to the amino acid in the ω position. The GPI-APs are then transported to their final destination via the Golgi system. During transport, further modification of the anchor lipids occurs in a process termed lipid remodeling (4). GPI-APs may be lost from the membrane either with their anchors intact, in a process termed shedding, or upon enzymatic cleavage, i.e., by phosphoinositol-specific phospholipases B and C (5) (see Fig. 1). Biosynthesis, biochemistry and cell biology, trafficking, organization, and dynamics at the cell surface and the release of GPI-APs have all been reviewed recently in greater detail (4, 6–11). To these detailed insights into the topic, we would like to add information about the applications of GPI-APs in biotechnology, and more specifically, in biomedicine (12–14). These applications are mainly based on the membrane-targeting properties of GPI-APs and directed at modifying or functionalizing lipid bilayer membranes. This can be achieved in two different ways: by genetic (genotypic) engineering (GE) or by protein engineering [PE, also termed phenotypic engineering, protein transfer, or molecular painting (MP)]. **Figure 2** summarizes the differences, advantages, and disadvantages of the two strategies. Hallmarks of the development of GPI-AP membrane engineering are depicted in **Fig. 3**.

GE introduces recombinant DNA containing the key sorting signals of N-terminal signal peptide and C-terminal GSS into suitable producing cell lines, e.g., via transfection or infection methods (15–17). GSS elements are commonly derived from the naturally GPI-anchored regulators of the complement system cluster of differentiation (CD)55 (18), CD59 (19), or the Fc-receptor CD16b (15). The choice of the GSS can determine the cell membrane compartment localization (20).The recombinant DNA constructs will express, modify, traffic, and finally display recombinant GPI-APs. The natural GPI-anchoring biosynthesis pathway is subverted to display the protein of interest. Generating such GPI-AP-producing cell lines is also a necessary prerequisite for purification of GPI-APs when conducting MP. Extracellular membrane vesicles produced from these cells will contain the recombinant GPI-APs (15, 17). When the GPI-producing cells are generating enveloped virus-like particles (VLPs), viruses, or viral vectors (VVs), GPI-APs will be included in the viral envelope (15, 21, 22) as a result of the colocalization of viral exit points and GPI-APs in membrane domains, i.e., lipid rafts (LRs) (13, 23, 24). Both reflect physiological sorting mechanisms that are employed to direct the GPI-APs to sites of relevance for biomedical application.

MP exploits a specific property of the GPI-Aps, which is the ability of purified GPI-AP preparations to spontaneously reinsert into lipid bilayer membranes (18, 25–33). Technically, the mixing and incubation of lipid bilayer membranes and a sufficient quantity of purified GPI-APs at 37°C is enough to initiate the process (followed by an optional removal of noninserted protein, i.e., by centrifugation; see Fig. 2). This property of GPI-APs was described for the first time in 1984 (see Fig. 3 for an overview of the development of GPI-AP membrane engineering) and is based on work by Medof, Kinoshita, and Nussenzweig (25). In this original study, the human regulator of complement CD55 (or decay-accelerating factor) was purified and found to be inserted into erythrocytes upon coincubation (see **Table 1** for an overview of proteins used for GPI-AP membrane engineering). The association was shown to increase in a time- and temperature-dependent manner and the protein’s original complement regulatory activity was conserved. Interestingly, the nature of the membrane anchoring of CD55 was not known at the time and only discovered 2 years later (34). Carrier lipids and small amounts of detergents seem to enhance the process (19, 20, 35, 36). Inter-cellular transfer of GPI-APs has also been described under physiological conditions: onto maturing sperm cells (37) or, for CD59, from erythrocytes to endothelial cells (38), as well as for trypanosomal variant surface glycoprotein, to erythrocytes of infected patients (39). However, mechanisms for these protein transfer events may vary and commonly involve extracellular lipid vesicles (40–42). Protein transfer processes employing such vesicles would also allow for a degree of specificity, mediated by ligands on the protein donor vesicle and receptors on the acceptor membranes. Very little is known about how MP occurs. Mechanistically, a process where small aggregates of GPI-APs, eventually augmented by carrier lipids or detergent, first fuse with lipid bilayer membranes and then diffuse laterally seems likely. This is supported by the fact that signaling properties of GPI-APs are not restored immediately upon MP. Alternatively, a mechanism involving endocytosis and internal insertion followed by redistribution has been discussed (43). Integration is directly mediated by the fatty acid residues of the GPI anchor. Enzymatic removal effectively abrogates the ability to insert (18) and insertion is poor at 4°C (25). Several routes toward clinical application for MP have been explored since then, including the use of recombinant GPI-anchored CD4 as a strategy for human immunodeficiency virus (HIV)-mediated gene therapy (44) and the use of the natural, non-recombinantly GPI-AP CD55 and CD59, to treat paroxysmal nocturnal hemoglobinuria (PNH) (45, 46). In PNH, a defect in GPI anchoring leads to an enhanced susceptibility to the complement system. Also, approaches toward tumor therapy and vaccination using GPI-anchored variants of the costimulatory molecule, B7.1 (47, 48), the cytokines, interleukin (IL)2 (49, 50) and IL12 (51–53), granulocyte/macrophage-colony stimulating factor (GM-CSF) (19), the human epidermal growth factor (EGF) receptor 2 (HER2) tumor antigen (36), and the intercellular adhesion molecule, (ICAM)1 (CD54) (19), were undertaken. The MP process was adapted for the modification of enveloped viral particles in 2008, originally employing lenti- and γretroviral particle-derived gene therapy vectors (28). Later the range of viral species was expanded to orthomyxo- and herpesviral particles, as well as the range of modifying proteins, to GPI-anchored variants of green fluorescent protein (GFP) (18), the red fluorescent protein tdTomato (54), EGF (55), the HIV receptor CD4 (55), and IL2 (49, 50). The ability of the virus or virus vector to infect is not necessarily hindered as a consequence of insertion (28). However, caution is advised because reductions in infectivity due to the presence of additional proteins on the envelope are possible under certain circumstances (29). The process is strictly dependent on the presence of the GPI anchor lipid parts, insertion increases with increasing amounts of virus and GPI-APs, and more than one protein may be inserted at the same time (18). Also membrane vesicles were shown to be modified by PE with GPI-APs (29, 35, 52). More information about recent studies will be provided in the following sections.

**TABLE 1.** GPI-APs used for membrane engineering

Protein	Function	Target Membrane	Application	Type	Comment	Reference
CD59	Complement protection	CE, BC, VE, MV	IM	GE, PE	Naturally GPI-AP	22, 28, 29, 45, 46, 58, 67, 77, 78, 81
CD55	Complement protection	CE	IM	GE, PE	Naturally GPI-AP	25, 32, 45, 46, 58, 67, 81
65kD-HRF	Complement protection	CE	IM	PE	Naturally GPI-AP	65
GFP	Fluorescent marker	CE, VE	LB	PE	Aggregation issues in VE	18, 20
mGFP	Fluorescent marker	VE	LB	PE		18
tdTomato	Fluorescent marker	VE	LB	PE		Unpublished observations
IL2	Cytokine	CE/AE	IM	GE, PE		15, 49, 50, 62, 86
IL4	Cytokine	VE	IM	GE		15
IL7	Cytokine	VE	IM	GE		15
IL12	Cytokine	MV	IM	PE		52, 53
IL15	Cytokine	VE	IM	GE		15
IL21	Cytokine	CE	IM	GE		16
GM-CSF	Cytokine	VE	IM	GE, PE		15, 19, 21
CCL28	Cytokine	VE	IM	GE		85
CXCL10/mucin	Chimeric cytokine	CE	IM	PE		31
GIFT4	Chimeric cytokine	VE	IM	GE	GM-CSF/IL4 fusion protein	87
IL2R	Cytokine receptor	VE	IM	GE		86
B7.1 (CD80)	Costimulatory molecule	CE, MV	IM	PE		35, 47, 48, 68, 71, 102
B7.2 (CD86)	Costimulatory molecule	CE	IM	PE		71
CD40L	Costimulatory molecule	VE	IM	GE		21
VEGF	Growth factor	VE	TG	PE	Aggregation issues	Unpublished observations
EGF	Growth factor	VE	TG	PE		55
Her2	Growth factor receptor	VE, MV	IM	PE	Displayed as tumor-associated antigen	35, 36
EPCR	Protein C receptor	CE	HR	PE		76
CD4	HTV receptor	CE	IM/TG	PE		44
ICAM 1	Adhesion molecule	VE	IM	PE		19
LFA3 (CD58)	Adhesion molecule	CE	IM	PE	Naturally GPI-AP	33
scFv	Antibody derivatives	VE/LUV	TG	GE	LUV-VLP fusion vesicle for delivery	88
Nanobodies	Antibody derivatives	EX	TG	GE	Specific for EGFR	17
Alkaline Phosphatase	Hydrolytic enzyme	CE/LP	R	PE	Naturally GPI-AP	98, 99
Acetylcholinesterase	Hydrolytic enzyme	CE	R	PE	Naturally GPI-AP	32, 66
C34	Entry inhibitor	CE	HR	GE		59
EPS_GPI_	Trypanosomal GPI proteins	LP	IM	PE		100
TIMP-1	ECM remodeling	CE	HR	PE	In wound healing and tumor therapy	31, 74, 75

CD, cluster of differentiation; CD40L, CD40 ligand; CE, cell membrane; EGF, epidermal growth factor; EPCR, endothelial protein C receptor; EPS, GPI-protein extract; EV, extracellular vesicle; GPI, glycosylphosphatidylinositol; Her, human epidermal growth factor receptor; HRF, homologous restriction factor; ICAM, intercellular adhesion molecule; IL, interleukin; IL2R, IL2 receptor; IM, immunomodulation; LB, labeling; LFA, lymphocyte function-associated antigen; LP, liposome; LUV, large unilamellar vesicle; MV, membrane vesicle; (m)GFP, (monomeric) green fluorescent protein; R, research ; scFv, single chain variable fragment; TG, targeting; TIMP, tissue inhibitor of matrix metalloproteases; VE, viral envelope; VEGF, vascular endothelial growth factor.

Modification of lipid membranes with GPI-APs may be employed for different goals: labeling, targeting, and manipulation of host responses (HRs), mostly for immunomodulation. Labeling may be mostly useful in research settings to follow the fate of cells, viruses, membranes, or membrane compartments such as LRs (18, 20). In biomedical settings, the labeling may enable monitoring of gene/cell therapy approaches, but also contribute to purification and/or concentration of virus or VLP preparations for gene therapy or vaccination. Additionally, emerging enveloped viruses, where limited biochemical information will make specific enrichment difficult, are targets for concentration or purification by PE. Targeting may facilitate a more efficient gene therapy (56), as well as enable a more directed immune response in vaccine development (57). HR makes use of ligand-receptor interactions to trigger desired cellular responses. In most cases, this will involve a manipulation of the immune system (51), either to stimulate (15, 19, 21, 31), i.e., by cytokines, or to inhibit (22, 29, 58), i.e., by delivery of complement regulatory factors such as CD55 or CD59.

## MODIFICATION OF CELLS

Both GE and MP approaches have been used to modify cellular membranes. Only recently, a GE approach was suggested in the field of HIV therapy (59). In this study, the GPI-anchored peptide, C34, which inhibits the entry of different HIV subtypes, was transduced into susceptible cell lines. The modified cells were then challenged with retroviral vector particles pseudotyped with various viral envelope glycoproteins (59) and infection was shown to be greatly diminished because C34 interferes with the fusion of viral envelope glycoproteins and cell membranes (60, 61) through action as a decoy of the viral gp41 fusion protein. Entry inhibition was at least as prominent as for the soluble C34 peptide indicating that the function of the inhibitor was not disturbed by the GPI tethering. Other studies employing GE on cells were conducted in the field of tumor immune therapy. In one case a plasmid construct encoding GPI-anchored IL2 was delivered by lipofection to the murine melanoma cell line, B16F0 (ATCC® CRL6322™) (62). When injected into mice, the growth of the IL2-modified tumor cells was inhibited, an effect that was not observed when administering soluble IL2 (62). In other studies a GPI-anchored version of the pleiotropic cytokine, IL21 (63), was employed either alone (16) or in combination with secreted GM-CSF (64) in a similar setting. Here B16F10 murine melanoma cells (ATCC® CRL6475™) were modified and investigated for their immune-stimulatory potential. When used as a tumor cell vaccine on mice, the modified cells were shown to reduce tumor size and prolong survival. Again, the GPI-anchored variant produced a stronger effect than the secreted IL21 (16). Also, the combination of GPI-AP IL21 and soluble GM-CSF showed better results than either of the compounds alone (64).

MP approaches are favored in cases where GE may be difficult, i.e., on erythrocytes that lack a nucleus. Indeed, in early studies on GPI membrane insertion, erythrocytes provided the target lipid bilayer (25, 65, 66). Regarding MP on cellular membranes, the GPI-APs may either be delivered to cells in vivo (30, 31) or ex vivo and eventually (re-)implanted. In parallel to gene therapy, the latter may prove to be the more efficient and practical approach. Often, early attempts at MP employed naturally occurring GPI-APs with a function in complement regulation, such as CD55 (25), CD59 (67), or the 65 kDa homologous restriction factor (65). These can help to alleviate the symptoms of PNH, a chronic disease characterized by loss of protection of cells from the complement system and subsequent hemolytic anemia (45, 46, 67). A second focus for MP of cells was developed early in applications for tumor therapy (31, 47, 53, 68–70). In 1995, a GPI-anchored variant of the costimulatory molecule, B7.1 (CD80) (see also Table 1), was introduced to different tumor cell lines, including the human breast cell carcinoma cell line T47D (ATCC® HTB-133™), the human melanoma cell line SKMEL28 (ATCC® HTB-72™), and the human lymphoblastic leukemia cell line MOLT4 (ATCC® CRL-1582™). GPI-B7.1-treated cells were able to initiate sufficient costimulatory signals to elicit stimulation of T cells (47). Similar approaches using B7.1 or B7.2. (CD86) produced from a different source on different tumor cell lines confirmed the results (68, 71). In these cases, the mechanism is mostly immunomodulation, i.e., in studies using cytokines. The chemokine, CXCL10, which recruits natural killer cells, was engineered to contain a GPI anchor in addition to a carbohydrate-rich mucin domain. While the first would allow the CXCL10 domain to integrate into cellular membranes, the latter would maintain chemokine function under physiological flow conditions (31). In vivo experiments confirmed the increased recruitment of natural killer cells, compared with control groups, upon direct delivery of the GPI-APs into the tumors. Other molecules related to tumor initiation or progression may also be used for therapeutic strategies, thus providing a flexible and versatile approach. The tissue inhibitor of matrix metalloproteases (TIMP)-1 (a regulator of extracellular matrix modulation with cytokine-like properties) (72, 73) was engineered to contain a GPI anchor and was delivered to different tumor cell lines by MP. GPI-TIMP-1 was shown to inhibit the growth of fibrosarcomas and enhance the tumor sensitivity to doxorubicin treatment, also in vivo, when delivered directly to the tumor (30), thus circumventing loss of efficacy due to unspecific insertion in non-tumor membranes. The reduction of tumor volumes was significantly increased for GPI-TIMP-1 treatment compared with soluble TIMP-1 or control treatments (30). The same molecule increased the sensitivity of melanomas and renal cell carcinomas to FAS-mediated apoptosis (69, 70). While both the mode of action (decreased flexibility of the extracellular matrix) and delivery (direct GPI-AP delivery to the tumors and on-site integration) seem feasible and in vivo mouse models yield promising data, further preclinical and clinical research is needed. Other medical conditions that may be targeted by GPI-AP membrane engineering include wound healing, again by using TIMP-1 (74, 75), or manipulation of the pro­tein C system involved in anti-coagulant and cyto-protective processes (76). Also, gram-negative bacteria have been modified by MP. Both *Escherichia coli* and *Helicobacter pylori* incorporated CD59, at least in one case in an anchor-dependent manner (77, 78). The protein remained functional and protected bacterial cells from complement lysis.

## MODIFICATION OF VIRUS PARTICLES

For viruses, the application of GPI-APs for membrane modification is limited to species carrying a lipid shell, the envelope, around their protein capsids. This phospholipid bilayer is derived from the host cell during budding. This includes the families retro- (HIV), orthomyxo- (influenza), flavi- (Zika, dengue), phyllo- (Ebola), herpes- (Epstein-Barr), and poxviridae (Variola). In this section, we will discuss the modification not only of enveloped virus particles, but also of VLPs and VVs used for gene therapy [collectively termed virus/derived particles (V/DPs)]. What these engineered variants have in common is that they lack important parts of the full viral anatomy: in VLPs, viral structural proteins are used to generate a lipid vesicle population of good homogeneity. They are mostly used as particulate antigen-presenting platforms (79). As a consequence, infection of or entry into cells is not always required and, in these instances, VLPs do not need to carry viral proteins mediating particle entry, thus increasing biological safety. In contrast, VVs are mostly used for the delivery of recombinant DNA. At least one round of viral entry to cells is necessary to deliver the genetic material; however, no virus production in the infected cell will be initiated as a consequence of viral genome engineering (80). The first suggestions to use GPI-APs for the modification of V/DPs were fueled by the observation that HIV and other viruses include GPI-APs into their envelopes, more specifically, the GPI-anchored regulators of complement activity, e.g., CD55 and CD59, as a means to protect themselves from their host’s immune response (22, 58, 81–83). Research into membrane sub-structures or domains, such as LRs, defined the mechanistic framework for these observations: GPI-APs and sites of viral budding may colocalize in LRs (13, 23, 24). Initial biotechnological applications were developed for gene therapy, using CD55 and CD59 to protect retroviral vectors from the complement system by GE (22, 58). The advent of lentiviral vectors produced in human cells for gene therapy applications made these approaches mostly obsolete, because the particles would contain natural CD55/CD59. However, the idea of using GPI-APs for the modification of V/DPs was upheld. GE approaches using cytokines or growth factors artificially anchored by GPI (15, 21) were used to facilitate vaccination approaches (15, 21, 84, 85) and promote immunological research by studying receptor-ligand interaction (86). Recently, GE approaches have been employed to facilitate vaccination approaches using a chimeric cytokine (87) and to support tumor therapy by targeting of colon cancer cells using single chain variable fragments (88). While the latter significantly broadens the application range of the technology by combining the diversity of recombinant antibody technology and the speed of GPI-AP membrane engineering, the earlier approach employs a chimeric cytokine (termed GIFT 4) consisting of IL4 and GM-CSF elements to enhance mucosal immunity against HIV-1. In vivo experiments on guinea pigs revealed higher levels of systemic antibodies with increased binding avidity and improved neutralizing properties (87). However, further testing is required.

In 2008, the first attempt at MP for the modification of V/DPs was published (28). CD59 was delivered to retro- and lentiviral vectors (28) and later shown to confer partial resistance to complement activity (29). Variants of GFP were used to modify lenti-, herpes-, and orthomyxovirus particles in a dose-dependent manner. Also, two independent GPI-APs (CD59 and GFP) could be associated with a lentiviral vector simultaneously (18). While these approaches were mostly targeted at facilitating gene therapy using VVs (18, 29, 55, 89, 90), recently strategies for the use in vaccination were suggested (19, 36, 51, 91, 92). In these studies, influenza VLPs were generated in a recombinant baculovirus system (93, 94) and modified, on one hand, with either GPI-anchored IL12, GM-CSF, or ICAM-1 (collectively termed GPI immunostimulatory molecules) as adjuvant agents in anti-viral immune responses (19) and, on the other hand, with GPI-HER2 as a model for protein transfer of a tumor-associated antigen for tumor vaccination (36). Both approaches demonstrated good stability of the insertion and elicited enhanced immune responses compared with untreated controls and were shown to be protective in animal experiments (19, 36). In the anti-tumor study, it was suggested that, as a result of the VLP association, both Th1- and Th2-type related antibody responses (i.e., subtypes of humoral immunity characterized by immunoglobulin subtype patterns) were triggered, opposed to the soluble form of the antigen, which mostly induced Th2 responses (36). This is especially interesting, because Th1-type responses play an important role in anti-tumor immunity (95). The anti-viral study demonstrated the flexibility of the system by associating several different molecules (GM-CSF, IL12, ICAM-1) separately (19). The possibility for displaying more than one GPI-AP simultaneously has already been demonstrated previously (18). Taken together, both studies indicate the potential for a highly versatile and flexible system for directing immune responses using different combinations of antigen and adjuvant molecules. Again, further research is necessary and should be encouraged.

## MODIFICATION OF OTHER LIPID VESICLES/SURFACES

One of the advantages of MP is that no metabolism is required for carrying out the functionalization. Thus, a wider range of lipid vesicles is available for modification, including liposomes, model membranes (96), and cell-derived membrane vesicles, such as exosomes. Also, nonlipid particles with hydrophobic characteristics are amenable to MP by GPI-APs, i.e., BSA particles (97). Liposomes can only be modified by PE (98–100) because GE of cellular sources is not applicable. Exosomes or other extracellular vesicles, however, may be functionalized by GE, as was demonstrated recently when displaying GPI-anchored nanobodies on extracellular vesicles after transfection of the murine brain cancer cell line, Neuro2A (ATCC® CCL-131™) (17). Nanobodies are antibody fragments consisting of a single variable domain and thus comprise the smallest antigen-binding structural unit (101). The diversity of nanobodies binding specifically [in this case to EGF receptor (EGFR)] should allow for efficient targeting of different tumor antigens by enhanced attachment (in this case increased binding of nanobody-targeted vesicles to the EGFR). Indeed, binding of vesicles displaying specific nanobodies to EGFR-positive A431 cells (ATCC® CRL-1555™) was approximately 10-fold increased, compared with unmodified or control-modified particles. Marker expression patterns and size distributions were not altered as a consequence of GE (17). This suggests that the original protein (i.e., antigen) background of the vesicle can still be exploited after GPI-AP modification, e.g., in tumor vaccination strategies.

Extracellular vesicles are also amenable to membrane modification by MP and may profit from the quick exchange facilitated by protein transfer. In a recent article, CD59 was attached to VVs, but protein was also retained in control samples containing concentrated supernatant from non-virus-producing cell lines (albeit at significantly reduced levels). It is most likely that exosomes are the target of modification because their accumulation would be favored by the preparation method (29). Also, membrane vesicles of different origin have been modified by MP with GPI-APs (35, 48, 52, 102). The most interesting approach in this area seems to be the use of tumor-derived membranes or vesicles (35, 102). Surgically removed tumor tissue can be used to generate membrane vesicles displaying the original tumor antigens. Such vesicles, in turn, can be modified by MP with immune stimulatory molecules, i.e., B7.1 (102), targeting molecules or additional tumor antigens, i.e., HER2 (35), to induce, direct, and/or increase anti-tumor immune responses. In the last approach, exogenous tumor antigen is introduced by MP to cell membrane vesicles (of an average diameter of approximately 330 nm) derived from experimentally generated tumors in mice. As shown in a similar study with influenza VLPs, both Th1 and Th2 responses were initiated (36). After a prime and boost vaccination regime, mice were protected from a challenge with tumor cells carrying HER2, indicated by a decrease in tumor area and a concomitant increase in tumor-free survival. Interestingly, the membrane vesicle antigenic background did not seem to play a role in inducing immunity (35). However, by including stimulatory factors, an effective immune response against the original tumor background can be mounted. Such approaches would combine the advantages of personalized therapy with the speed of GPI-AP membrane engineering and the efficacy of anti-tumor immune therapy. Further preclinical research is also indicated in this area.

## DISCUSSION, SUMMARY, AND CONCLUSIONS

There are several issues regarding the use of GPI-APs in biomedicine/biotechnology that warrant closer consideration. For example, expression levels seem to be lower than those achieved with the parental protein (unpublished observations). This is most likely a consequence of the complex biochemistry of the GPI-anchoring metabolic pathway. In order to provide sufficient amounts for clinical testing, several options may allow increasing expression levels, including the induction of GPI metabolizing enzymes by zinc ions (49) and overexpression of the enzymes of GPI biosynthetic pathways by “metabolic engineering.” However, due to the complexity of the GPI metabolism, this may prove challenging. Additionally, the use of alternative expression systems, i.e., the leishmania-based LEXSY (https://www.jenabioscience.com/lexsy-expression), may help to overcome bottlenecks in GPI-AP production. Parasite cells (i.e., *Trypanosoma* or *Leishmania sp.*) are especially rich in GPI-APs, i.e., the trypanosome variant surface glycoproteins, which cover the whole cell (103). Some issues only apply to MP, i.e., challenges surrounding the purification of GPI-APs. Challenges associated with the purification of membrane proteins, such as achieving efficient and gentle solubilization, also apply to GPI-APs. Additionally, the chemical sensitivities of the lipid anchor need to be taken into consideration, i.e., avoiding alkaline treatment to prevent saponification. The inherent strong hydrophobic nature of GPI-APs also makes aggregation an issue. As a consequence, it is vital to use a non-membrane control alongside the samples in MP processes to assess multimerization or aggregation issues, especially when dealing with nano-sized vesicles such as viral particles or exosomes, which are more likely to be “contaminated” by similarly sized aggregates, which is less of an issue when working with cells (18). Additives may help to overcome these issues: although not necessary, the use of small amounts of detergent (i.e., n-octyl-β-D-glucopyranoside) or carrier lipids (i.e., cholesterol) can promote PE (19, 20, 35, 36). Generally, the amount of time the GPI-APs spend outside of the (cell) membrane may reduce function and/or transfer efficiency. Because the insertion process is only membrane-targeted: systematic in vivo delivery of GPI-APs is problematic. For example, the anchor will not differentiate normal cells from tumor cells. Also, inhibition of insertion by lipid-transfer proteins, such as albumin or different types of lipoproteins, will reduce transfer yield (43). For this reason, either ex vivo approaches or the use of particulate carriers, such as VLPs, vector cells, or liposomes, seems promising. Multiple proteins can be inserted into such carriers providing targeting functions.

Another open issue is the vice versa influence of the two alternative aspects in GPI-APs: how the hydrophilic protein and the lipophilic fatty acids may influence each other and the GPI-AP performance in biomedical applications. Parameters of interest include protein size and composition, as well as the exact chemical composition of the GPI anchor. While little data is available about the latter, protein size is known to influence diffusion behavior of GPI-APs (103). The reduced lateral motility of larger polypeptides may also have a negative influence on MP applications. Additionally, the larger protein part may reduce the maximum density of insertion due to steric hindrance. Although systematic data is lacking, we could only achieve a very limited insertion of the approximately 70 kDa large CD55 (decay-accelerating factor), when compared with GPI-anchored GFP (35 kDa) and CD59 (18 kDa) (unpublished observations). Also, the removal of the GPI anchor can change protein structure and thus function and/or antigenicity (104). Thus, it stands to reason that addition of a GPI anchor may also influence protein function. Addition of flexible peptide linkers may help to overcome such issues.

In spite of the technical challenges, there are some inherent advantages of GPI-AP applications in general, such as efficient membrane targeting (also into derived vesicles), the small (and therefore less disturbing) footprint in the membrane, and a degree of mechanical/structural flexibility due to the GPI linkage, making it less likely to lose soluble protein function. Other advantages are specific to MP. These advantages include the speed of modification (hours compared with days because no expression is necessary) and the fact that time consuming or difficult GE can be completely avoided (i.e., for certain cell lines or patient-derived cells). In some cases, where GE is impossible (i.e., for virus particles of unknown genetics, patient-derived extracellular vesicles, or liposomes), MP may be the only option. Additionally, the level of insertion is controllable, multiple GPI-APs can be inserted by MP (18) and target membranes from differing sources, i.e., different tumor cells or different virus species (18) can be modified with the same GPI-AP.

Finally, chemical alternatives to membrane targeting, including lipophilic carbocyanine dyes such as DiI (1,1′-dioctadecyl-3,3,3′,3′-tetramethylindocarbocyanine perchlorate) or DiO (1,1′-dilinoleyl-3,3′-oxacarbocyanine perchlorate) (105), for labeling purposes, and function-spacer-lipid constructs (106, 107) are available. However, they may lack the inherent biocompatibility of GPI-APs, both in terms of attaching functional proteins to the compounds and in delivery to cells, vesicles, or virus membranes. Such products have reached the stage of commercialization (see http://www.kodebiotech.com/), as well as compounds based on GPI-APs (see http://www.metaclipsetherapeutics.com/ and http://www.anovasia.com/)

In summary, a wide range of proteins have been GPI-anchored by recombinant DNA techniques and used for biomedical purposes to date (see Table 1 for an overview). Both GE and PE (see Fig. 2 for comparison) is employed for the modification of cell membranes and derived vesicles, as well as viruses and derived particles. In terms of clinical applications, gene therapy, immune therapy, tumor therapy, vaccination, and combinations of these fields are favored and the most promising areas of development for strategies employing GPI-APs in membrane engineering.

Future clinical application is only a step away, and it seems most feasible to employ a platform vesicle-based formulation (i.e., exosomes, tumor membrane vesicles, enveloped viral particles) in (most likely tumor) vaccination strategies, making use of combinations of antigens and stimulatory molecules, as well as using the inherent bio-compatibility of patient-derived membranes (52). In conclusion, the collected set of publications strongly indicates the potential and efficacy of GPI-APs for a range of biomedical applications. Development of techniques facilitating their use, both by GE and MP, should be encouraged.
